# Genito-Urinary Surgery

**Published:** 1904-09-10

**Authors:** 


### Progress in Medicine and Surgery.

### GENITO-URINARY SURGERY.

(*Continued from page* 394.)

Hydronephrosis.—B. G. A. Moynihan[7] relates two cases in which hydronephrosis was cured by plastic operations. The first patient, a woman of 19, had had pain in the right loin since two years of age, and four weeks before operation a swelling had formed in the lumbar region. At the first operation the relation of the ureter to the pelvis of the kidney was found to be abnormal, the former beginning at the junction of upper third with the lower two-thirds of the pelvis. Also the ureter was stenosed at its point of origin, and a probe could not be passed through the stricture. The stricture was divided longitudinally and sutured horizontally. About six weeks later the hydronephrotic swelling had formed again, and at a second operation the ureter was cut completely across, and the distal end sutured into the lowest part of the renal pelvis. This cured the patient. The stenosis of the ureter was probably congenital, and its high opening caused by the earlier and greater distension of the lower part of the pelvis. The second case was that of a woman of 21 who first had trouble in the left side after a confinement, and a swelling formed there. At the operation the cause of the hydronephrosis was found to be a valve-like formation at the junction of the ureter and the renal pelvis. This was divided longitudinally and sutured transversely. A cure resulted. Three months later, with the Luys segregator, it was found that the patient possessed only one functionating kidney viz., the left one, which had been operated upon The case shows the importance of conservative treatment for hydronephrosis. If the patient's solitary kidney had been much injured by the operation death would have been inevitable. Paul Delbet,[8] in a paper on paranephric cysts and hydronephroses following traumatism, states that of 46 ca es 63·2 per cent. belonged to the former category and 36·8 pe[illegible]
cent. to the latter. The paranephric cysts arise i[illegible]
about 5 per cent. of renal contusions. They form lar[illegible]
swellings which contain pure urine, or blood and uri[illegible]
or urine and pus. The kidney lies at the back of [illegible]e
swelling, and the ureter runs along the inner [illegible]f
anterior wall. The interior of the cavity may [illegible]n-
municate either with the ureter or pelvis of [illegible]id
kidney. For the production of traumatic hy[illegible]e.
nephrosis there must be some obstruction to the
flow along the ureter, such as impacted blood
stricture, obliteration of the lumen, compressi[illegible]N.
peri-ureteral bands, or kinking of the u[illegible]
Laceration of the renal parenchyma is not su[illegible]with
to produce a paranephric cyst; in fact, the sur[illegible] uro-
a tear in renal tissue does not excrete. It is com-
to account for these cysts, for the urine is not[illegible]nxiety
under any pressure. Delbet considers that [illegible]e goes
factor is infection with the colon bacill[illegible]and of
prevents healing of the wound in the [illegible] effects
ureter and destroys the resistance of the [illegible]o cause
tissues. The diagnosis between the two[illegible]be borne
swelling described is often difficult or i[illegible]tiseptic,
Points in favour of a paranephric cyst are,[illegible]ly large subject; appearance of the swelling early, *i.e.* in three or four weeks; absence of violent pains at the time the tumour appears, and of a sudden stoppage of hæmaturia. A tumour which remains unaltered for weeks or months is most probably a hydronephrosis. In treatment it is best to make a lumbar incision. Nephrectomy may then be performed if the kidney is much disorganised, or the ureter may be dealt with by suture, uretero-plasty, anastomosis, or separation of adhesions. James Hamilton[9] records the case of a man of 22 who was operated upon for a left hydronephrosis. The patient died from peritonitis and anuria on the fourth day. At the autopsy the right kidney was found to be also hydronephrotic. It is remarkable that with such a marked congenital condition of both sides the man should have lived so long and been in such good health until within a few weeks of his death.

**Intravesical Separation of the Urines from the two Ureters.**—R. A. Bickersteth[10] has written on this subject, especially with reference to the use of the Luys separator. It is frequently of great importance to determine the functional efficiency of each kidney. A kidney should never be removed unless it has been ascertained that its fellow is present and in good working order. The author regards catheterisation of the ureters for the purpose of collecting samples of the urine from each kidney as a method of procedure too complicated, difficult, and troublesome to form part of an ordinary routine examination. The separation of the urines can be much more easily effected with the separator of G. Luys, of Paris, or that of Cathelin, also of Paris. With both these instruments a vertical water-tight septure is expanded in the bladder, dividing its cavity into two lateral halves corresponding to the two ureters. The urine from each half of the bladder is drained away by catheter-tubes and collected in receptacles externally. The Luys separator is used with the patient sitting upright. The instrument has to be gently pressed upon the floor of the bladder after the partition has been expanded by turning a screw. For the comparative examination of the few centimètres of urine obtained the quantity of urea in each specimen may be estimated with a Little's ureameter, and the freezing-point may be determined. Also, if methylene blue be administered previously, the degree of coloration of each specimen will be in proportion to the renal efficiency.

**Cystoscopy.**—Follen Cabot[11] has devised a cystoscope of a simple kind which can be used for observation by direct view, for irrigation and for catheterisation of both ureters at the same time. The catheters lie in the bottom of the sheath of the instrument and the telescope at the top. The catheters lie in a common chamber, except for half an inch at the point of entrance. The instrument is easy to clean and easy to withdraw, leaving the catheters in the ureters. It is made by the Wappler Electric Controller Company of New York. Louis E. Schmidt,[12] in a paper on cystoscopy, wrote that he believed there were but few, if any, authentic cases of infection of the ureter following catheterisation. Catheterisation of the ureters and injection of sterilised liquid vaseline had several times been efficient in causing the expulsion of stones from the ureters into the bladder, and from the latter cavity they had subsequently escaped *per urethram*. In performing a plastic or other operation on the renal pelvis, and in operations for abdominal tumours or inflammatory processes, it was a help to have a bougie passed into the ureter as a guide. Also catheterisation was useful for draining the pelvis of the kidneys in pyelitis and for flushing out that cavity and treating it with nitrate of silver or other solutions. A. Galbraith Faulds[13] advocates the use of oxygen for distending the bladder for cystoscopy instead of fluid media. This has been rendered possible by the introduction of the so-called "cold lamps" for cystoscopes, which give an excellent light with little or no heat. Faulds has experimented with air and gases, purifying them before passing them into the bladder, and has found that he can get a much better view by using oxygen than by any of the other media tried. The gas is passed in from a rubber bag containing the amount of oxygen required, and provided with a vulcanite cock which fits into the mouth of the catheter. In chronic cystitis oxygen ameliorates the condition and has a clarifying effect on the urine. The gas must be pure and be allowed to enter the bladder slowly. When cystitis exists, a local anæsthetic will have to be used. The gas may be left in the bladder without evil effects and often with advantage.

**Tumours of the Bladder.**—C. B Lockwood[14] has recently discussed the early diagnosis and the treatment of bladder tumours. Such tumours are usually overlooked until operation offers little chance of cure. The malignant tumours make their way very slowly through the bladder wall, but they project into the cavity of the bladder. There appear to be very few, if any, lymphatics in the mucous coat of the bladder, but this does not apply to the prostate. Many malignant growths of the bladder have a pedicle. The first sign of a tumour is often spontaneous hæmorrhage at the end of micturition. The next symptoms are the results of septic infection of the bladder, usually from instrumentation. Statistics are quoted by the writer to show how much higher the mortality of operations for the removal of bladder tumours becomes if the bladder is previously septic. In simple papilloma of the bladder the hæmorrhage is very irregular, and there may be intervals of 12 or 18 months without hæmorrhage. These intervals, however, become shorter and shorter, until at last there is bleeding with every act of micturition. Statistics of St. Bartholomew's Hospital show that the proportion of malignant to innocent tumours is four to one. If a tumour of the bladder can be felt above the pubes or per rectum or per vaginam it is inoperable. The endoscope renders the early diagnosis of bladder tumours very simple. In removing these tumours it is essential to see what you are doing, and the tumour itself should not be cut into but should be removed with a wide margin of healthy tissue. For the subsequent drainage of the bladder Guyon's double-way drainage tube, which can act as a syphon, is of great service. L. W. Reynolds[15] records the case of a man of 60 from whom he removed villous tumours of the bladder. At the operation several velvety prominences, partially pedunculated, and two larger growths were found, the latter in the trigone. The two larger growths and about a dozen smaller pieces were removed. Nine months later there had been no recurrence.

[7] Brit. Med. Jour., April 1904, p. 1010. [8] Abst. Med. Chronicle, March 1904, p. 405. [9] Glasgow Med. Jour., February 1904, p. 120. [10] Lancet, March 26, 1904, p. 859. [11] Med. Record, March 26, 1904, p. 520. [12] Abst. Med. Record, March 12, 1904, p. 437. [13] Brit. Med. Jour., March 5, 1904, p. 539. [14] Lancet, June 11. 1904, p. 1633. [15] Brit. Med. Jour., April 2, 1904, p. 782.

(*To be continued.*)

